# Cigarette Smoking Among Socioeconomically Disadvantaged Young Adults in Association With Food Insecurity and Other Factors

**DOI:** 10.5888/pcd13.150458

**Published:** 2016-01-14

**Authors:** Jin E. Kim, Janice Y. Tsoh

**Affiliations:** Author Affiliation: Janice Y. Tsoh, University of California, San Francisco, Department of Psychiatry, San Francisco, California.

## Abstract

**Introduction:**

Low socioeconomic status is associated with high rates of cigarette smoking, and socioeconomic differences in cigarette smoking tend to emerge during young adulthood. To further our understanding of socioeconomic differences in smoking among young adults, we examined correlates of smoking, with attention to multiple socioeconomic indicators that have not been examined in this population.

**Methods:**

We analyzed data from the 2011–2012 California Health Interview Survey. The analytic sample consisted of young adults aged 18–30 years who were considered socioeconomically disadvantaged as measured by education and poverty. Logistic regression analyses were conducted to examine factors associated with smoking status in this group, and multinomial logistic regression analyses were conducted to examine correlates of smoking frequency.

**Results:**

In this sample (N = 1,511; 48% female, 66% Hispanic/Latino, 18% non-Hispanic white), 39.7% reported experiencing food insecurity in the past year. Smoking prevalence was significantly higher among young adults who reported being food insecure (26.9%) than among those who reported being food secure (16.4%). Past-year food insecurity was significantly associated with current smoking, independent of sociodemographic characteristics and alcohol use. Specifically, food insecurity was significantly associated with daily but not nondaily smoking.

**Conclusion:**

Socioeconomically disadvantaged young adults with food insecurity may be considered a high-risk group with respect to cigarette smoking. Efforts to reduce tobacco-related health disparities should address diverse sources of socioeconomic influences, including experiences of food insecurity.

## Introduction

Low socioeconomic status (SES) is associated with high rates of cigarette smoking (1). These socioeconomic differences tend to emerge during young adulthood (2); young adults who have not attended or are not enrolled in college smoke at twice the rate (30%) of their college-educated counterparts (14%) (3). Furthermore, smoking cessation is a challenge, as young adults underuse evidence-based cessation treatments (4) and often engage in social or nondaily smoking (5). Smoking contributes to socioeconomic inequities in disease and mortality (6), and the socioeconomic context of smoking in young adults has been understudied.

One potentially important variable is food insecurity, or the lack of physical and economic access to adequate and appropriate foods needed to live an active and healthy life (7). In 2014, food insecurity affected 40% of US households living in poverty and 14% of households overall (7). Food insecurity is associated with many negative health consequences and overall poorer health (8). The association between food insecurity and cigarette smoking was previously documented, with findings showing higher smoking prevalence among members of low-income families with past-year food insecurity (44%) than their food-secure counterparts (32%) (9). Another study reported that food insecurity among households with at least one adult smoker (26%) was higher than among households with no adult smokers (12%) (10).

This study examined the association of food insecurity with smoking and its patterns (daily and nondaily smoking) among socioeconomically disadvantaged young adults. We adjusted for sociodemographics and behavioral health factors, psychological symptoms, and alcohol use, which are often associated with higher smoking rates among young adults (11,12). We used data collected from a representative and diverse sample of young adults aged 18 to 30 years residing in California and examined those considered to be at risk for food insecurity based on indicators of SES such as education and poverty.

## Methods

### Data set and sampling

We used publicly available data from the 2011–2012 California Health Interview Survey (CHIS) (13). CHIS is the largest population-based state health survey in the United States, assessing health and demographic information from noninstitutionalized California residents. It uses a multistage sampling design and random-digit–dialed (landline and cellular) telephone numbers. CHIS has been conducted every other year since 2001. The 2011–2012 CHIS Adult Survey had 42,935 adult respondents, and approximately 14% of the interviews were completed in a language other than English. More detailed information regarding the survey is available (13). The sample for our study had 1,511 young adults aged 18 to 30 who were socioeconomically disadvantaged as measured by education (ie, highest educational attainment of grade 12 or below) and poverty (ie, annual income below 200% of the federal poverty level [FPL]). FPL is set annually by the US Department of Health and Human Services and is used to assess eligibility in federal assistance programs, such as Medicaid and the Supplemental Nutrition Assistance Program. The 2011–2012 CHIS used poverty guidelines from 2007, which included an annual income of $10,210 for a 1-person household and $20,650 for a 4-person household living in the contiguous United States (14). Because the study data were de-identified and were available to the public, the University of California San Francisco’s institutional review board did not require formal study approval, but rather, documented self-certification.

### Measures

Two questions assessed current smoking status and frequency: “Altogether, have you smoked at least 100 or more cigarettes in your entire lifetime?” and “Do you now smoke cigarettes every day, some days, or not at all?” Current smokers were considered to be those who had smoked 100 or more cigarettes in their lifetime and also currently smoked every day (daily smokers) or some days (nondaily smokers). Nonsmokers were former smokers (people who had smoked ≥100 cigarettes in their lifetime but did smoke currently) and people who never smoked regularly (had not smoked ≥100 cigarettes in their lifetime and did not smoke currently).

Past-year food insecurity was assessed via the US Household Food Security Survey, 6-item short form (15). In the CHIS, the food insecurity module was administered to all individuals who were below 200% of FPL (study inclusion criteria). The short form is a validated subset of 6 items from the 18-item Food Security Survey developed by the US Department of Agriculture and included in the Current Population Survey (7,15). Food insecurity was determined by at least 2 affirmative responses to the 6 items (eg, “In the last 12 months, did you ever eat less than you felt you should because there wasn’t enough money to buy food?”) (16).

Past-year psychological distress was assessed by the 6-item Kessler Psychological Distress scale (K6) (17). The K6 measured nonspecific depressive and anxiety symptoms such as hopelessness and worthlessness experienced in a 30-day period on a scale of 0 (none of the time) to 4 (all of the time). The 6 items are summed to create a distress score ranging from 0 to 24, with a score of 13 or more indicating severe psychological distress. On the basis of the K6 score for the worst month in the past year, past-year psychological distress was categorized into 3 levels: none/mild (scores of 0–4), moderate (scores of 5–12), and severe distress (scores of 13–24) by using published cutoff scores derived from 2007 CHIS data (18).

Two questions assessed any past-year alcohol use (yes or no) and past-year binge drinking (≥5 alcoholic drinks in a single day for men or ≥4 alcoholic drinks in a single day for women). On the basis of these 2 questions, we formed 3 alcohol use categories in reference to the past year: no alcohol use, alcohol use without binge drinking, and alcohol use with binge drinking.

Sociodemographic characteristics were age (categorized as 18–25 y and 26–30 y based on previous literature on young adulthood) (19), sex, race/ethnicity (African American, Asian American, Hispanic/Latino, non-Hispanic white, and other), nativity and English proficiency (categorized as US-born, foreign-born but English proficient, and foreign-born with limited English proficiency), highest educational level (categorized as grade ≤8, grade 9–11, and grade 12 or equivalent), poverty (categorized as 0%–99% or 100%–199% of FPL), and having a usual source of health care (categorized as yes or no).

### Statistical analyses

We conducted weighted descriptive analyses to derive population-level frequencies on variables of interest across smoking status. We conducted a multiple logistic regression analysis to examine factors associated with current smoking status and a multinomial logistic regression analysis to examine factors associated with smoking frequency (daily or nondaily smoking) by using nonsmoking as the reference group in both cases. We categorized race/ethnicity as non-Hispanic white, Hispanic/Latino, and other because of small sample sizes of smokers in certain racial/ethnic groups. All analyses were conducted in SAS version 9.4 (SAS Institute, Inc) using CHIS final sampling weights to estimate parameters, and we used replicate weights and the jackknife replication method to estimate standard errors (20).

## Results

The analytic sample included 1,511 young adults considered to be socioeconomically disadvantaged (<200% FPL and ≤12 years of education) (Table 1). By smoking status, 20.5% were current smokers, and 79.5% were nonsmokers (9.5% were former smokers, and 70.0% never smoked regularly). Approximately 40% reported past-year food insecurity; food insecurity rates were higher among current smokers than nonsmokers. Smoking prevalence was significantly higher among young adults who reported being food insecure (26.9%) than among those who reported being food secure (16.4%).

**Table 1:** Weighted Sample Characteristics, Socioeconomically Disadvantaged Young Adults Aged 18 to 30 Years, by Smoking Status, 2011–2012 California Health Interview Survey

Characteristic	Total Sample		Current Smoker		Nonsmoker^a^		
	N (Unweighted)	% (95% CI)	n (Unweighted)	% (95% CI)	n (Unweighted)	% (95% CI)	*P* b
**Total sample**	1,511	100.0	299	20.5 (17.4−23.7)	1,212	79.5 (76.3−82.6)	—
**Age, y**							
18–25	1,070	70.2 (67.0−73.4)	192	64.3 (56.5–72.1)	878	71.7 (68.0–75.4)	.09
26–30	441	29.8 (26.7−33.0)	107	35.7 (27.9–43.5)	334	28.3 (24.7–32.0)	
**Sex**							
Male	709	52.3 (48.8–55.8)	174	65.9 (48.8–73.1)	535	48.8 (44.7–52.9)	<.001
Female	802	47.7 (44.2–51.2)	125	34.1 (29.9–41.2)	677	51.2 (47.1–55.3)	
**Race/ethnicity**							
African American	77	5.2 (3.8–6.6)	16	5.1 (1.6–8.5)	61	5.3 (3.7–6.8)	<.001
Asian American	117	8.7 (6.4–11.0)	10	5.0 (0.0–10.9)	107	9.7 (7.2–12.2)	
Hispanic/Latino	938	66.4 (63.2–69.5)	138	51.9 (43.9–60.0)	800	70.1 (66.6–73.6)	
Non-Hispanic white	330	17.5 (14.6–20.4)	114	33.4 (25.1–41.6)	216	13.4 (10.7–16.0)	
Other	49	2.2 (1.0–3.4)	21	4.7 (0.1–9.2)	28	1.6 (0.7–2.5)	
**Nativity and English proficiency**							
US-born	1,007	67.1 (63.9−70.4)	236	76.3 (68.7–84.0)	771	64.7 (61.0–68.5)	.03
Foreign-born withEnglish proficiency	219	12.3 (10.2−14.5)	28	9.25 (4.6–13.9)	191	13.1 (10.7–15.5)	
Foreign-born with limited English proficiency	285	20.6 (17.9−23.3)	35	14.4 (8.5–20.4)	250	22.2 (19.1–25.2)	
**Highest educational level**							
Grade 8 or less	133	10.8 (8.7−12.8)	14	5.2 (1.4–9.0)	119	12.2 (9.7–14.7)	.002
Grade 9–11	267	21.6 (18.2−25.0)	69	32.3 (23.1–41.6)	198	18.8 (15.2–22.4)	
Grade 12 or equivalent	1,111	67.6 (64.4−70.8)	216	62.5 (53.4–71.6)	895	68.9 (65.3–72.6)	
**Poverty, % federal poverty level**							
0–99	879	55.8 (52.4−59.2)	183	56.5 (48.2–64.8)	696	55.6 (51.6–59.6)	.85
100–199	632	44.2 (40.8−47.7)	116	43.5 (35.2–51.8)	516	44.4 (40.4–48.4)	
**Have usual source of health care**							
Yes	954	60.3 (55.9−64.8)	165	51.5 (42.4–60.7)	789	62.6 (47.9–67.3)	.02
No	557	37.7 (34.2−41.3)	134	48.5 (39.3–57.6)	423	37.4 (32.7–42.1)	
**Alcohol use,^c^ past year**							
No alcohol use	630	39.9 (36.4−43.4)	50	18.6 (10.5–26.7)	580	45.4 (41.2–49.6)	<.001
Yes, with no binge drinking	352	24.4 (21.2−27.5)	68	28.3 (21.4–35.2)	284	23.3 (19.9–26.8)	
Yes, with binge drinking	529	35.7 (32.3−39.2)	181	53.1 (45.1–61.1)	348	31.2 (27.3–35.2)	
**Psychological distress, past year**							
None/mild	905	58.0 (54.2−61.7)	132	48.6 (39.4–57.8)	773	60.4 (56.2–64.5)	.01
Moderate	536	37.9 (34.1−41.6)	135	44.2 (35.3–53.1)	401	36.2 (32.2–40.3)	
Severe	70	4.2 (2.9−5.4)	32	7.2 (3.8–10.6)	38	3.4 (2.1–4.6)	
**Food insecurity status, d past year**							
Food secure	946	60.3 (56.4−64.1)	151	48.1 (39.4–56.8)	796	63.4 (58.9–67.9)	.002
Food insecure	564	39.7 (35.9−43.6)	148	51.9 (43.2–60.6)	416	36.6 (32.1–41.1)	

Abbreviations: CI, confidence interval; —, not applicable.

a Nonsmokers are former smokers and those who have never smoked regularly.

b *P* value derived from Rao-Scott χ^2 ^test.

c Binge drinking was defined as 5 or more alcoholic drinks in a single day for men or 4 or more alcoholic drinks in a single day for women.

d The lack of physical and economic access to adequate and appropriate foods needed to live an active and healthy life (7).

Food insecurity had a significant bivariate association with current smoking (odds ratio [OR], 1.87; 95% confidence interval [CI], 1.25–2.28). In the adjusted model, respondents with any past-year food insecurity had a 54% increase in the odds of current smoking compared with respondents without past-year food insecurity (Table 2). Significant sociodemographic characteristics in the multivariable model were age, sex, and race/ethnicity. Young adults aged 26 to 30 had higher odds of being current smokers than those aged 18 to 25. Women were about half as likely as men to be current smokers. Hispanics/Latinos and those in the “other” racial/ethnic group category had lower odds of being current smokers than non-Hispanic whites. Any alcohol use in the past year, compared with no alcohol use, increased the odds of current smoking. Furthermore, those smokers who binge-drank had a nearly threefold increase in the odds of current smoking relative to nondrinkers. Severe psychological distress was significantly associated with current smoking in the unadjusted analysis (OR, 2.66; 95% CI, 1.35–5.22), but the effect was no longer significant in the adjusted model.

**Table 2:** Logistic Regression Analyses of Smoking Status and Smoking Frequency, in Reference to Nonsmoking (N = 1,511), 2011–2012 California Health Interview Survey

Characteristic	Model 1: Smoking Status^a^ — Current Smoker		Model 2: Smoking Frequency^b^			
			Daily Smoker		Nondaily Smoker	
	AOR (95% CI)	*P* c	AOR (95% CI)	*P* c	AOR (95% CI)	*P* c
**Age, y**						
18–25	1 [Reference]					
26–30	1.75 (1.16−2.63)	.007	2.61 (1.61−4.21)	<.001	0.97 (0.47−2.01)	.94
**Sex**						
Male	1 [Reference]					
Female	0.52 (0.34−0.81)	.003	0.40 (0.23−0.67)	<.001	0.81 (0.49−1.35)	.42
**Race/ethnicity**						
Non-Hispanic White	1 [Reference]					
Hispanic/Latino	0.35 (0.24−0.53)	<.001	0.23 (0.14−0.38)	<.001	0.79 (0.40−1.52)	.50
Other	0.44 (0.20−0.96)	.038	0.56 (0.24−1.34)	.196	0.23 (0.09−0.58)	.002
**Nativity and English proficiency**						
US-born	1 [Reference]					
Foreign-born with English proficiency	0.90 (0.42−1.94)	.792	0.57 (0.23−1.41)	.224	1.61 (0.60−4.31)	.34
Foreign-born with limited English proficiency	0.66 (0.32−1.36)	.260	0.25 (0.09−0.74)	.012	1.89 (0.71−4.86)	.21
**Highest educational level**						
Grade 12 or equivalent	1 [Reference]					
Grade 8 or less	0.68 (0.27−1.70)	.404	1.37 (0.38−4.92)	.634	0.33 (0.08−1.31)	.12
Grade 9–11	1.83 (0.99−3.40)	.056	2.82 (1.34−5.91)	.006	0.85 (0.38−1.91)	.70
**Poverty, % federal poverty level**						
100–199	1 [Reference]					
0–99	1.20 (0.79−1.81)	.395	0.94 (0.57−1.55)	.808	1.62 (0.88−2.98)	.12
**Have usual source of health care**						
Yes	1 [Reference]					
No	1.32 (0.85−2.04)	.212	1.37 (0.79−2.39)	.265	1.19 (0.70−2.02)	.53
**Alcohol use, d past year**						
No use	1 [Reference]					
Yes, with no binge drinking	2.55 (1.36−4.78)	.004	2.39 (1.09−5.26)	.030	3.15 (1.18−8.38)	.02
Yes, with binge drinking	2.95 (1.61−5.39)	<.001	2.80 (1.32−5.94)	.008	3.41 (1.40−8.30)	.007
**Psychological distress, past year**						
None/mild	1 [Reference]					
Moderate	1.40 (0.90−2.16)	.133	1.22 (0.67−2.21)	.517	1.63 (0.96−2.76)	.07
Severe	1.37 (0.58−3.24)	.478	0.85 (0.25−2.99)	.795	2.64 (1.08−6.43)	.03
**Food insecurity status, e past year**						
Food secure	1 [Reference]					
Food insecure	1.54 (1.04−2.30)	.032	1.91 (1.67−3.12)	.010	1.02 (0.59−1.74)	.95

Abbreviations: AOR, adjusted odds ratio; CI, confidence interval.

a Reference group was nonsmokers (former smokers and those who never smoked) (n = 1,212). Model 1 was a multiple logistic regression model examining current smoking (n = 299) as the outcome.

b Reference group was nonsmokers (n = 1,212). Model 2 was a multinomial logistic regression model with outcome in 3 categories: daily smoker (n = 174), nondaily smoker (n = 125), and nonsmoker (n = 1,212).

c *P* value derived from Rao-Scott χ^2 ^test.

d Binge drinking was defined as 5 or more alcoholic drinks in a single day for men or 4 or more alcoholic drinks in a single day for women.

e The lack of physical and economic access to adequate and appropriate foods needed to live an active and healthy life (7).

Among current smokers, 63.3% (95% CI, 55.0–71.7) were daily smokers and 36.7% (95% CI, 28.3–45.1) were nondaily smokers. Respondents with any past-year food insecurity had a 91% increase in the odds of daily smoking compared with respondents without past-year food insecurity (Table 2). Food insecurity was not associated with nondaily smoking. Moreover, compared with nonsmokers, the odds of being a daily smoker were greater among young adults aged 26 to 30, those with 9 to 11 years of education, those who reported past year alcohol use with and without binge drinking, and those who experienced past year food insecurity. In reference to nonsmokers (Table 2), the odds of being a daily smoker were lower for women, Hispanics/Latinos, and foreign-born individuals with limited English proficiency. The odds of being a nondaily smoker were greater for young adults who reported past-year alcohol use with and without binge drinking and for those who reported severe psychological distress in the past year compared with nonsmokers.

## Discussion

In this representative sample of socioeconomically disadvantaged young adults aged 18 to 30 residing in California, approximately 40% of the sample reported household food insecurity in the past year. We found that smoking prevalence was significantly higher among those who reported food insecurity than among those who did not report food insecurity. Food insecurity increased the odds of current smoking, even when controlling for alcohol use and the demographic correlates of age, sex, and race/ethnicity. Our findings shed additional light on aspects of socioeconomic deprivation that may be related to higher smoking prevalence in disadvantaged populations. Namely, the experience of food insecurity was associated with both smoking status and smoking frequency among young adults with low SES.

The high prevalence of food insecurity in the study sample is consistent with previously reported rates among general households living in poverty (7). The high smoking prevalence among food-insecure young adults is also in line with prior research on smoking rates among members of low-income families with food insecurity (9). Our finding that food insecurity significantly raised the odds of current smoking adds new insight to a potential pathway between food insecurity and smoking in this population, given that we adjusted for a mental health correlate shared by smoking and food insecurity (21), namely psychological distress.

Many smokers report smoking for stress relief, whether or not it is effective. As with previous studies using similar data (22), our results showed a significant bivariate association between severe psychological distress — a specific form of stress — and smoking. However, the effect of severe psychological distress on the likelihood of current smoking was no longer significant in the adjusted model that included food insecurity. Stress is also one of the reasons for the positive association between poverty and smoking, because poverty gives rise to stressful conditions of living via a host of factors that often includes unmet subsistence needs, such as inadequate food. We might speculate that psychological distress is exacerbated by food insecurity (23), and the distress from being food insecure promotes smoking for stress relief. This possibility is consistent with existing cross-sectional evidence suggesting that, on the one hand, people smoke to relieve stress associated with financial strain, yet on the other hand, these smoking-related expenditures contribute to financial stress, particularly among the poor (24). These results lead us to hypothesize a similar reciprocal effect to be examined in future work, such that food insecurity increases the likelihood of smoking, but in turn, smoking worsens food insecurity among those living in or near poverty.

In terms of patterns of smoking, food insecurity was associated with daily smoking but it was not associated with nondaily smoking. These findings may point to the role of hunger in the association between food insecurity and nicotine dependence, because daily and nondaily smoking patterns often indicate different levels of nicotine dependence. Nicotine, the addictive psychoactive substance in tobacco, is an appetite suppressant that is associated with decreased food intake (25). One laboratory study showed that young adult female smokers who restrain their eating because of weight concerns used cigarettes as a means to prevent food intake when presented with a food prime (26). It is possible that smokers who are food insecure may be using cigarettes as a way to routinely (eg, daily) control their levels of hunger. Another possibility is that food insecurity may be predisposing nondaily smokers to eventually becoming daily smokers, because nondaily smokers become reinforced over time about the effects of nicotine on hunger. Future studies with prospective designs are needed to test these possibilities. Moreover, disadvantaged smokers tend to have greater nicotine dependence relative to nondisadvantaged smokers for numerous reasons, including differential exposure to stress (27). Although our data are not sufficient to assess nicotine dependence, the current findings raise future research questions about the role of food insecurity and its correlates, such as stress and hunger, in potentially contributing to progression in patterns of smoking.

Our findings also suggest age-related variations in patterns of daily and nondaily smoking among young adults in the current study. Daily smokers, compared with both nonsmokers and nondaily smokers, were more likely to be 26 to 30 years of age, whereas age was not significantly associated with nondaily smoking. Although most people try their first cigarette during their youth, it is often during young adulthood that most tend to either quit or become regular smokers (28). Our findings may be interpreted to suggest that the progression to daily smoking occurs later in young adulthood, and the extent to which food insecurity is associated with this progression remains to be studied. The sociodemographic factors that were significantly associated with smoking in this study, such as sex and race/ethnicity, are not necessarily novel findings but rather parallel much of the tobacco use literature. Given this consistency, examining the extent to which food insecurity influences cigarette smoking in other groups that disproportionately use tobacco is warranted.

Alcohol use was significantly associated with both smoking status and smoking frequency. Most young adults in our study reported drinking alcohol in the past year, with higher smoking prevalence reported among drinkers than nondrinkers. Social smoking, which is a common pattern of smoking among young adults (5), has been previously associated with increased co-use of alcohol (29). Though our data cannot speak to the co-use of alcohol and cigarettes per se, our results suggest that better understanding smoking behavior in the context of alcohol use is warranted in this population, and they suggest directions for future research.

We acknowledge limitations of our study. Measuring SES among young adults can be challenging, and we note that education and poverty levels are proxy measures that do not necessarily take into account other potentially informative indicators, such as subjective social standing and social mobility, among others. The cross-sectional nature of these data does not allow us to infer causality, but rather we demonstrated an independent association between food insecurity and cigarette smoking in this particular population. Mechanisms that link food insecurity and smoking remain to be examined in future investigations. Our study assessed any food insecurity at the household level in reference to the past year, which does not provide information about the severity or chronicity of food insecurity. In addition to cigarette smoking, future studies should investigate other types of tobacco and nicotine-delivering products that are becoming popular among young adults. We assessed smoking frequency as daily and nondaily smoking, because assessing other patterns of smoking, such as social smoking, was beyond the scope of the study given the availability of the data.

Our investigation is the first to our knowledge to establish food insecurity as a factor associated with current cigarette smoking status and smoking frequency among a diverse, representative sample of socioeconomically disadvantaged young adults in California. Even after controlling for sociodemographic characteristics and behavioral health correlates of smoking, food insecurity continued to have a significant independent effect on smoking status and smoking frequency. This initial evidence has implications for research on further understanding socioeconomic risk factors for smoking initiation as well as factors that may impede cessation among young adults and other populations with low SES. A more comprehensive and multidisciplinary approach is needed to reduce the disproportionately high rates of tobacco use among disadvantaged populations. Findings from this investigation draw attention to food insecurity as one aspect associated with socioeconomic context that may contribute to tobacco-related health disparities. Cigarette smoking and food insecurity are independently associated with chronic illnesses that pose serious threats to health, and understanding the health impact of both in conjunction should be an important area of research to reduce health disparities. These findings are relevant to public health policy research to reduce both smoking prevalence and food insecurity in disadvantaged groups. Further explorations of nontraditional venues, such as food assistance venues, may be warranted in research on smoking cessation interventions targeting socioeconomically disadvantaged young adults.
